# Supplementary material for: A landscape of complex tandem repeats within individual human genomes
Source: Nat Commun. 2023 Sep 14;14:5530. doi: 10.1038/s41467-023-41262-1 (PMC10502081; doi:10.1038/s41467-023-41262-1)
Supplement: Supplementary file 1 — Supplementary Information [file 41467_2023_41262_MOESM1_ESM.pdf]

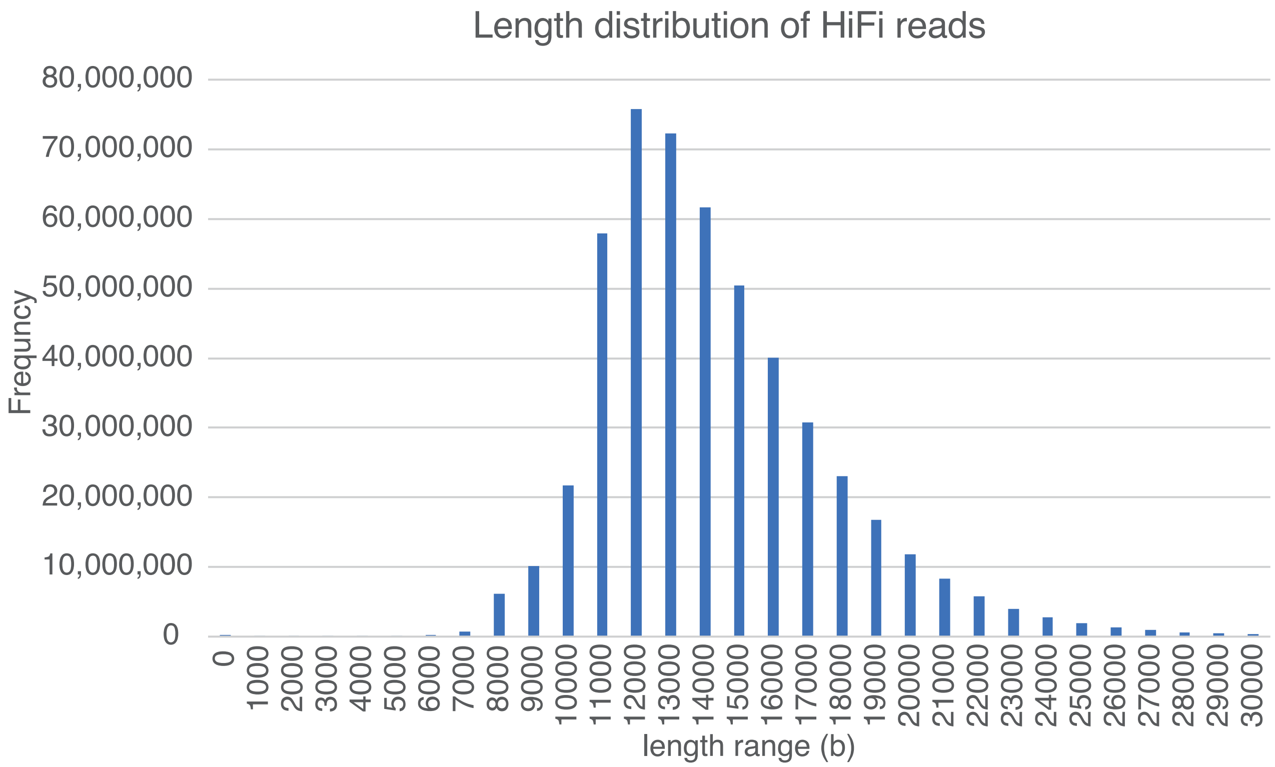

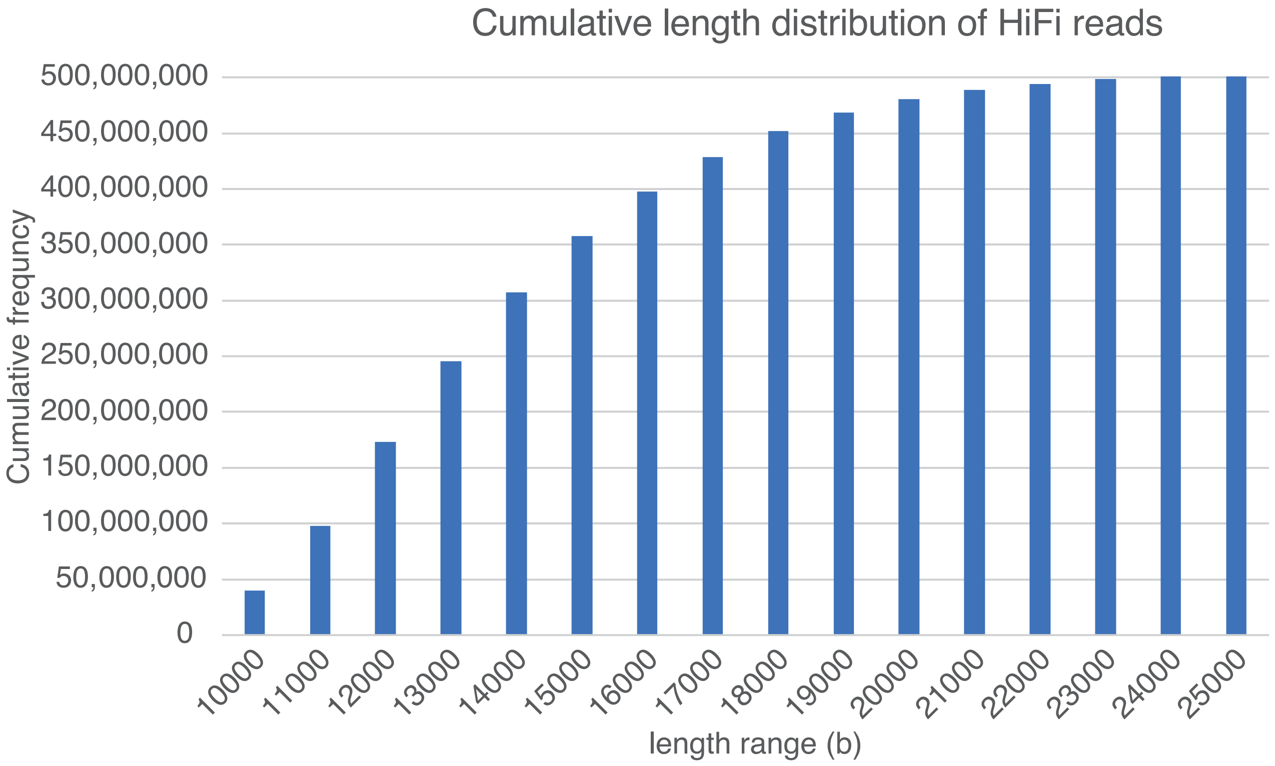

Supplementary Fig. 1: The length distribution of all PacBio HiFi reads that are used in this study. In the histogram above, each number on the x-axis represents a range from itself to the right-hand side minus one. The histogram below shows the cumulative frequency, which is the sum of the frequencies.

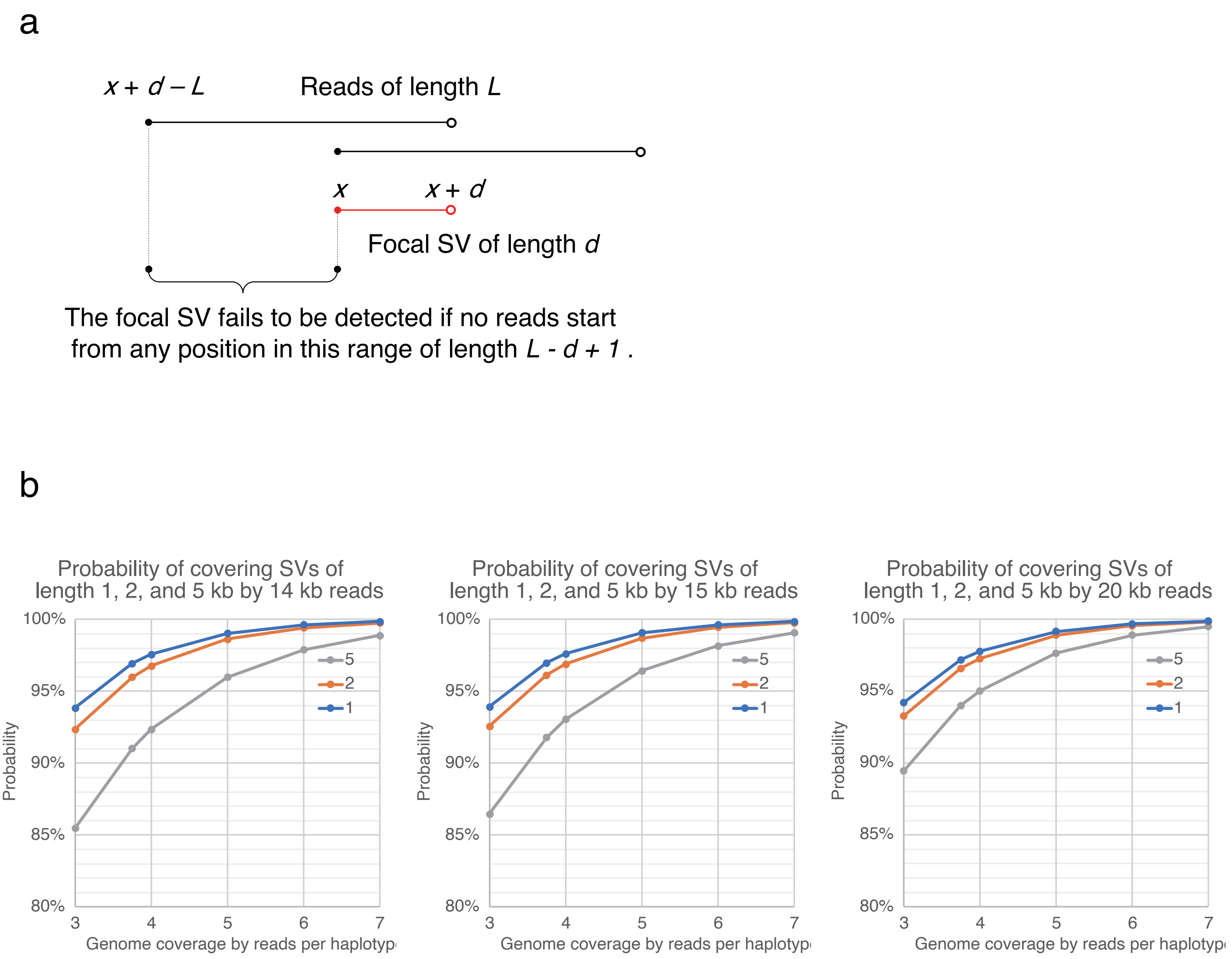

Supplementary Fig. 2: Probability of covering long SVs by long reads. a. The figure shows that reads of length L is able to cover the focal SV (red). **b.** Probability of covering SVs of length 1 kb (blue), 2 kb (orange), and 5 kb (gray) by reads of 14 kb (left), 15 kb (middle), and 20 kb (right) in size according to the Lander-Waterman statistics. The x-axis shows the genome coverage by reads per haplotype. We highlighted the coverage of 3.75 per haplotype because 3.75 is the half of 7.5 that is the minimum genome coverage of HiFi reads that we collected for each individual.

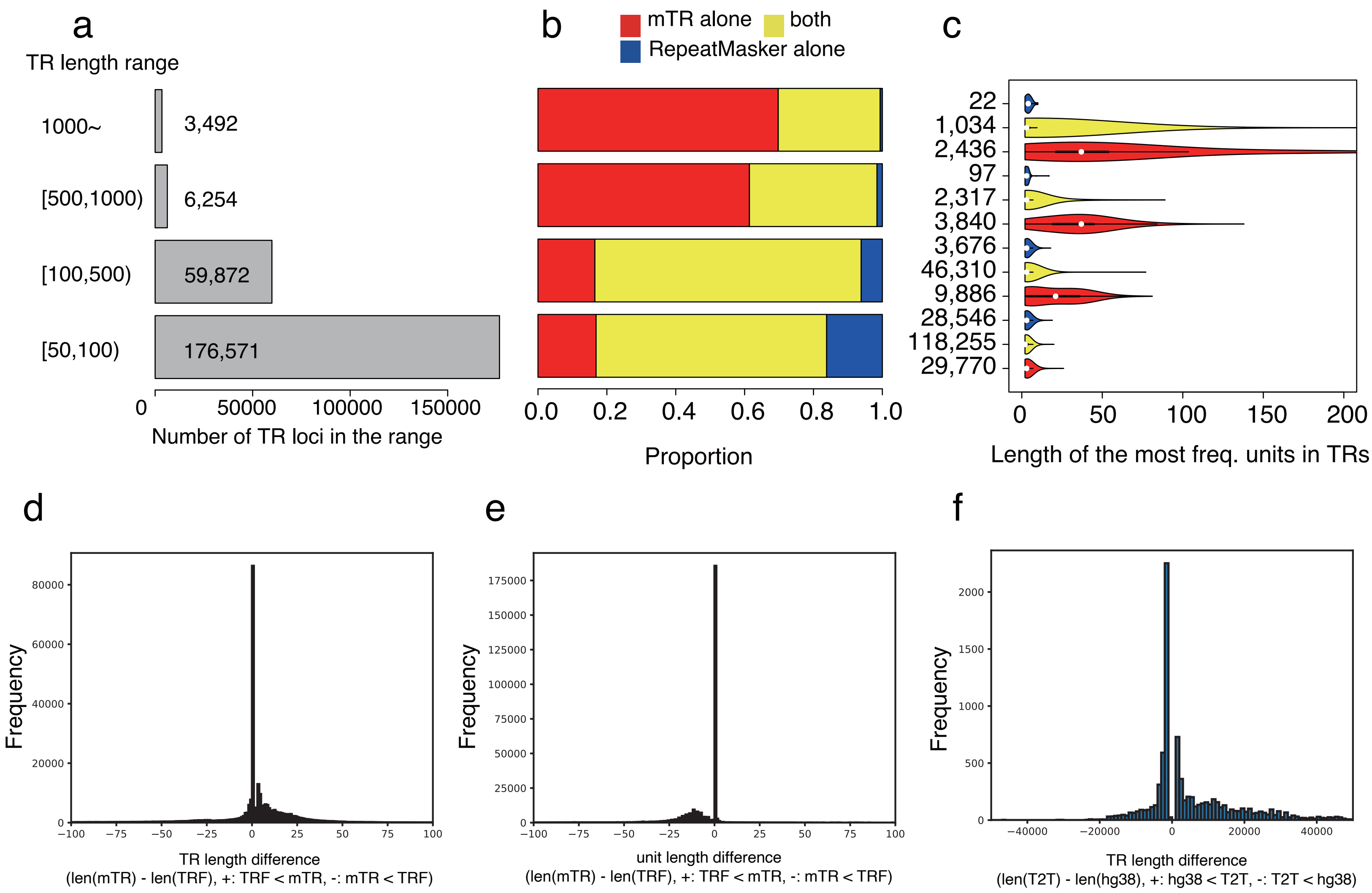

Supplementary Fig. 3: Finding tandem repeats in the reference genome. a, The length distribution of TRs in the human reference genome (hg38). In the left column, [50,100), for example, indicates the range from 50 b to 99 b (one before 100), and 1000~ the range from 1000 b. The number to the right of each bar indicates the exact number of TRs in it. **b,** Classification of each length range into three groups: TR loci are detected by mTR alone (red), RepeatMasker alone (blue), or both (yellow). The bars show the proportions of the three groups in each range. **c,** Because TRs at individual loci can have one or more units, the violin plots show the length distribution of the most frequent units in TRs. The left column shows the numbers of TR loci that are in the TR length ranges in Figure a and in the groups in Figure b. The color coding of the groups is the same as in Figure b. **d,** The histogram shows the distribution of differences in TR lengths estimated by mTR and TRF at the same TR loci. Zero indicates that both mTR and TRF estimate TRs of the same length, and plus (minus, respectively) indicates that mTR outputs TRs that are longer (shorter) than TRF. The first, second, and third quartiles are -8, 0, and 8, indicating that mTR and TRF estimate TRs of approximately the same length. **e,** Similarly, the unit lengths of TRs predicted by mTR and TRF are compared. The histogram shows the distribution of differences in TR unit lengths at the same TR loci. Zero, plus, and minus values are defined in the same way. The first, second, and third quartiles are -13, 0, and 0, showing that mTR is likely to estimate shorter units than TRF. **f,** Using Winnomap, TR loci in the hg38 reference genome were associated with their corresponding TR loci in the T2T reference genome, and ~1.97 million pairs of TR loci were found. Of these, 9,023 pairs differed by more than 1,000 nt in length and were further analyzed. The histogram shows the frequency distribution of length difference between TR loci in the hg38 reference genome and in the T2T reference genome. TR loci in the hg38 genome were likely to be shorter than their corresponding loci in the T2T genome.

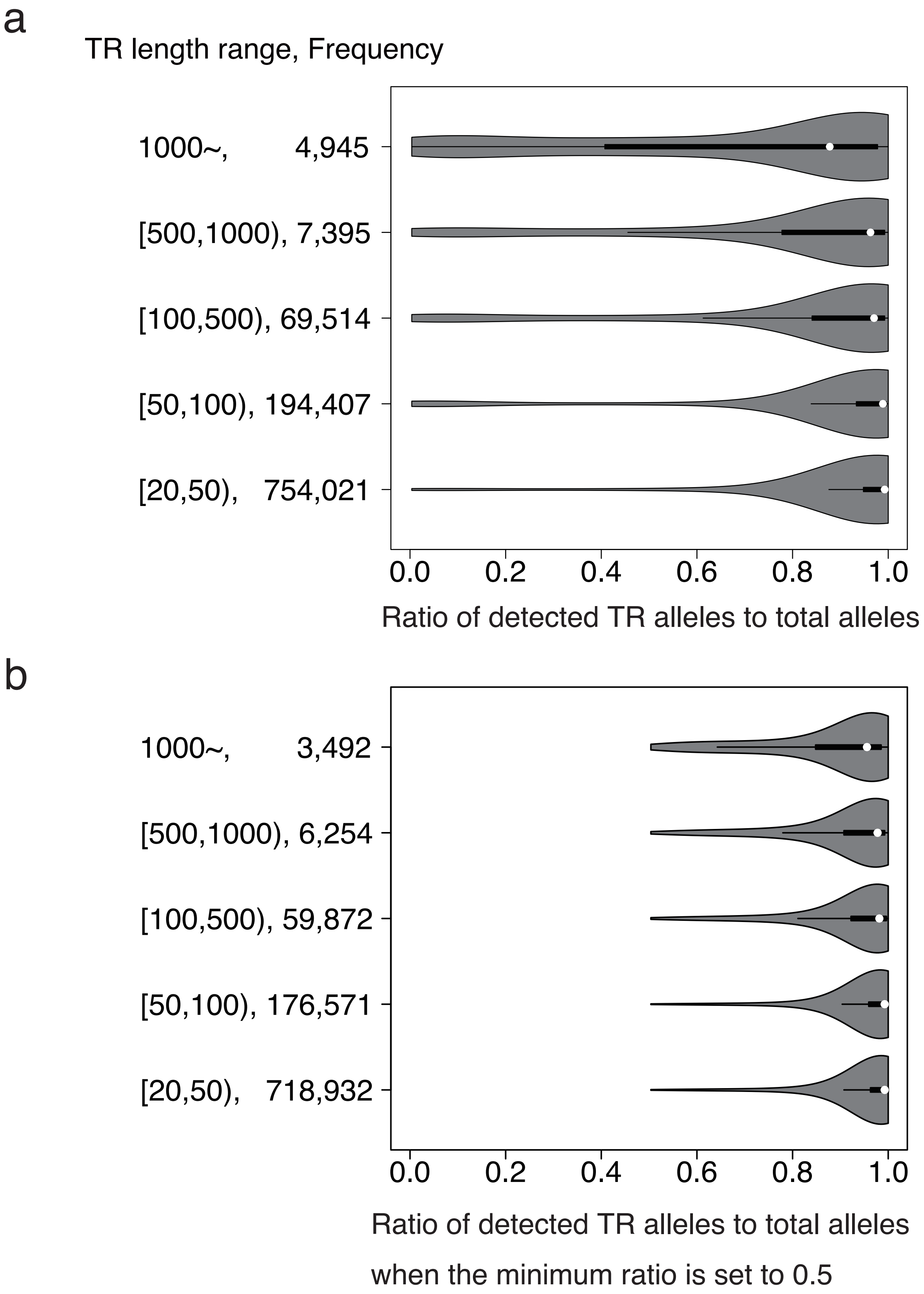

Supplementary Fig. 4: The distribution of ratio of the number of detected TR alleles to the total number of alleles with a doubled sample size at all TR loci. a, The distribution of ratios of 270 independent individuals is classified according to median length of TR alleles. The violin plot for each length range displays the ratio distribution. The white circle represents the median and the black thick line represents the lower and upper quartiles. **b,** Distribution of ratios when ratios are restricted to 0.5 or greater.

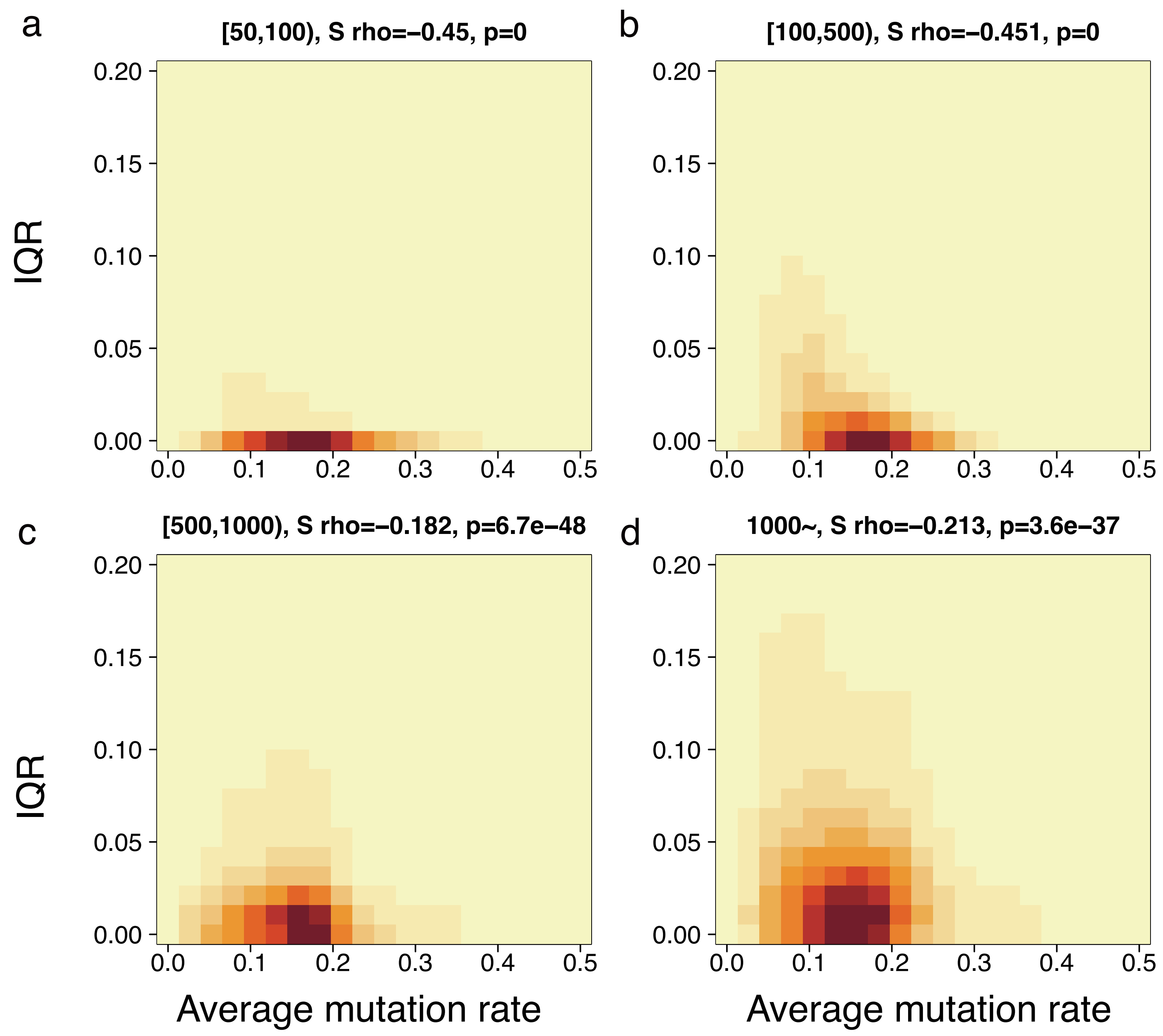

Supplementary Fig. 5: IQR correlates with average mutation rate. a-d, Kernel density estimates for two-dimensional data calculated from TR loci such that the lengths of TR loci are in the range [50,100) (**a**), [100,500) (**b**), [500,1000) (**c**), or 1000~ (**d**), the x-axis represents the average mutation rate, and the y-axis represents the IQR. The "S rho" and p in the title of each plot shows the Spearman's rank correlation coefficient and its p-value (two-sided Spearman's rank test). The correlation coefficients are all negative and the extremely small p-values indicate that they are statistically significant.

a

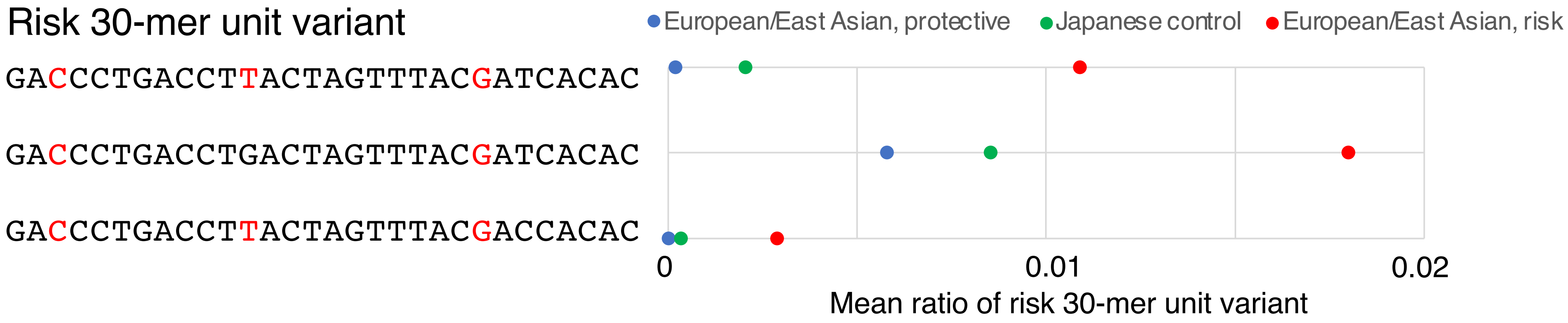

b

		TR alleles in Japanese individuals in this study					
	Number of TR alleles in 270 samples	11	11	4	4	2	2
	TR length	5610	5640	7770	5849	4620	5460
	Number of the 30-mer unit in TR	187	188	259	195	154	182
Count of risk units	GACCCTGACCTTACTAGTTTACGATCACAC	3	3	2	3	3	3
	GACCCTGACCTGACTAGTTTACGATCACAC	4	3	6	3	5	3
	GACCCTGACCTTACTAGTTTACGACCACAC	1	1	0	0	1	1
Fraction of risk units	GACCCTGACCTTACTAGTTTACGATCACAC	**0.0160**	**0.0160**	0.0077	**0.0154**	**0.0195**	**0.0165**
	GACCCTGACCTGACTAGTTTACGATCACAC	0.0214	0.0160	**0.0232**	0.0154	**0.0325**	0.0165
	GACCCTGACCTTACTAGTTTACGACCACAC	**0.0053**	**0.0053**	0.0000	0.0000	**0.0065**	**0.0055**

Supplementary Fig. 6: Tandem repeats with copies of three risk 30-mer unit variants that are correlated with bipolar disorder and schizophrenia at chr12: 2255791-2256090 in the human reference genome (hg38). a, Mean ratios of the occurrences of each risk unit variant to the total number of 30-mer unit occurrences in our Japanese control samples (green) and in the European/Asian individuals that are either risk (red) or protective (blue). The European/Asian data are from Song, J. H. T., et al. Characterization of a Human-Specific Tandem Repeat Associated with Bipolar Disorder and Schizophrenia. *Am. J. Hum. Genet.* **103**, 421–430 (2018). The mean of each variant in the Japanese controls is much smaller than that in the risk group of European/Asian individuals and are larger than that in the protective groups. **b**, Analysis of three risk 30-mer unit variants that occur in six TR representatives (in the right six columns) in the Japanese population. The 2nd to 4th rows show features associated with each TR representative: the number of TR alleles in it, its length, and the number of all 30-mer units in it. The 5th to 7th rows display the count of each risk unit variant in individual TR representative. The 8th to 10th rows the ratio of the count to the number of all 30-mer units in TR representative. Bold type indicates ratios that are greater than the mean plus standard deviation (0.0134, 0.0228, and 0.0044 for each of the three risk unit variants) in the risk individuals.

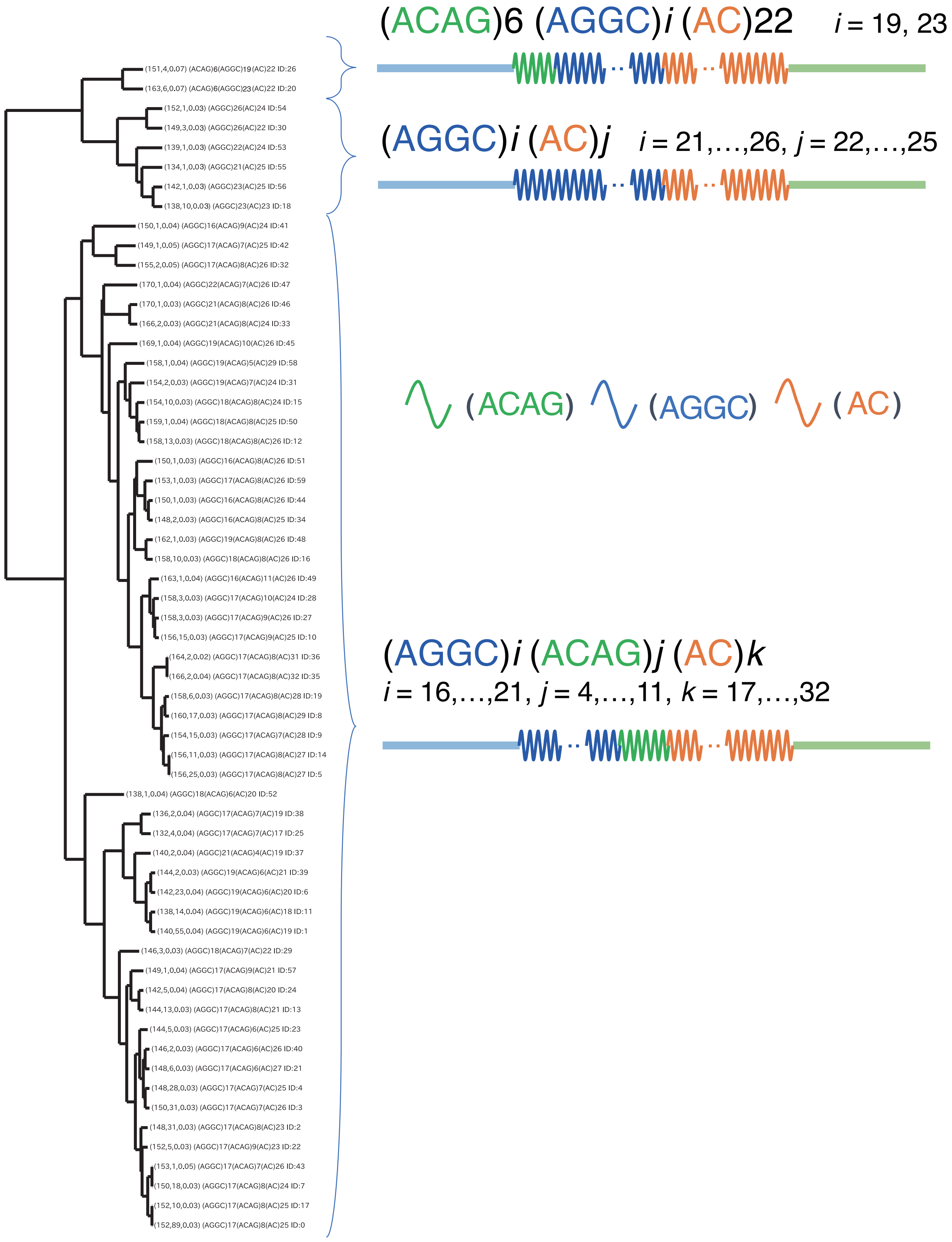

Supplementary Fig. 7: Phylogenic analysis of divergent TRs. Similar to Figure 5, the left phylogenetic tree shows the evolution of complex tandem repeats in an intron of *CNBP* at chr3:129,172,576-129,172,656 in the human reference genome (hg38), and the right illustrates several tandem repeat patterns with different units represented by colored waves. In the tree on the left, for example, the bottom TR representative (152,89,0.03) (AGGC)17(ACAG)8(AC)25 has 89 TR alleles in its members and is the most frequent representative.

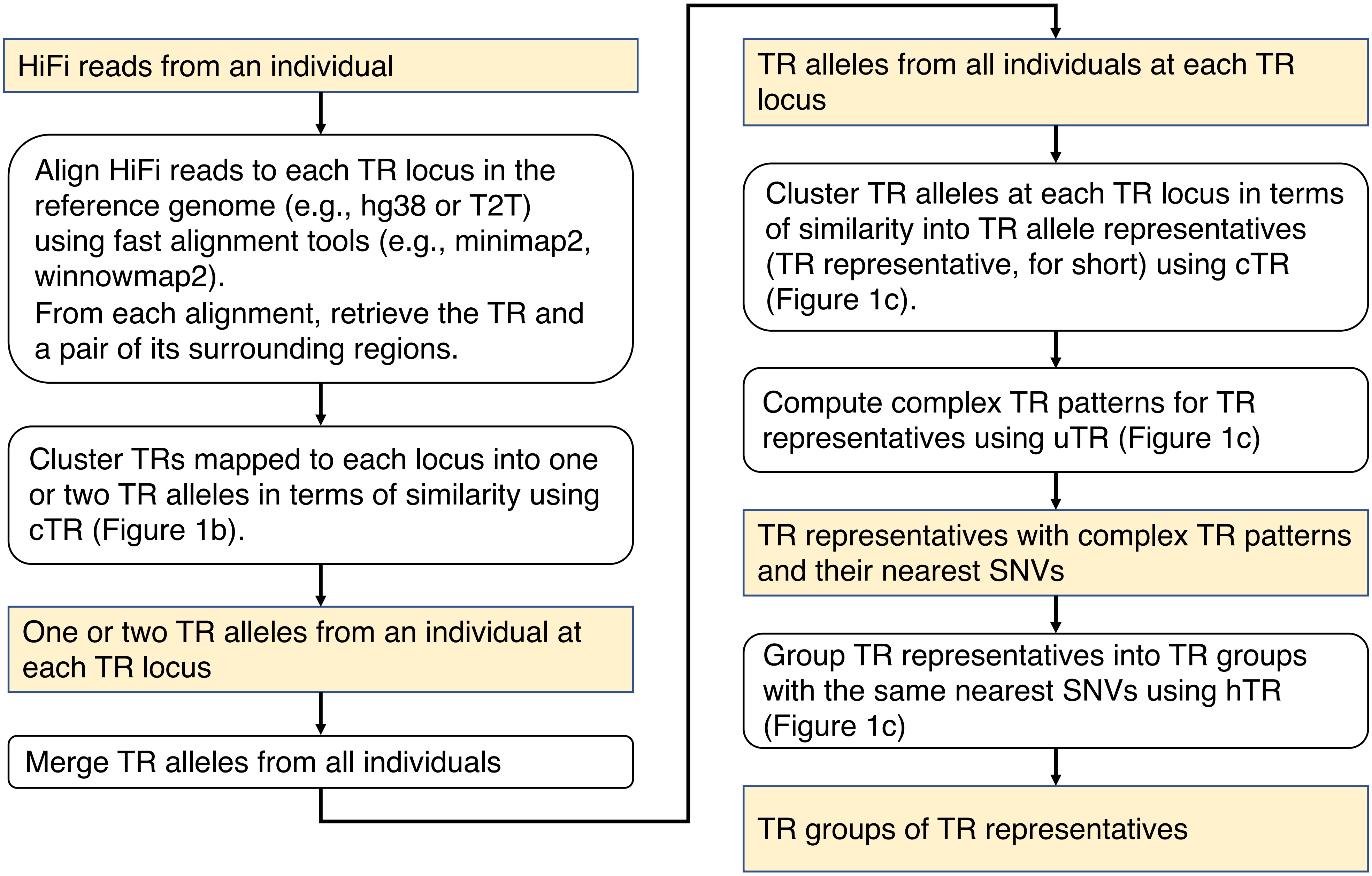

Supplementary Fig. 8: Flow chart outlining the different analysis stages. Yellow boxes represent input and processed data. Circled boxes outline the procedures and software used to handle the data.
