# Supplementary material for: A landscape of complex tandem repeats within individual human genomes
Source: Nat Commun. 2023 Sep 14;14:5530. doi: 10.1038/s41467-023-41262-1 (PMC10502081; doi:10.1038/s41467-023-41262-1)
Supplement: Supplementary file 3 — Description of Additional Supplementary Files [file 41467_2023_41262_MOESM3_ESM.pdf]

File Name: Supplementary Data 1

Description: The table shows TR loci such that the longest TRs were >100 bp longer than the median. If a TR locus in a gene coding region, we annotated it with the gene name and its location within the gene (exon, intro, UTR, etc) using the UCSC hg38 tables (see Methods).

File Name: Supplementary Data 2

Description: The table shows disease-associated TR regions in the GnomAD table (https://gnomad.broadinstitute.org/short-tandem-repeats?dataset=gnomad_r3) that are annotated by our program uTR. The data in columns A-E are obtained from the above URL, the data in column F from https://stripy.org/database, and the data in column G from Stevanovski et al. 2022 (PMID: 35245110). The data in columns H-I are annotated by our program uTR in this study.
