# Supplementary material for: A landscape of complex tandem repeats within individual human genomes
Source: Nat Commun. 2023 Sep 14;14:5530. doi: 10.1038/s41467-023-41262-1 (PMC10502081; doi:10.1038/s41467-023-41262-1)
Supplement: Supplementary file 6 — Reporting Summary [file 41467_2023_41262_MOESM6_ESM.pdf]

nature portfolio

Corresponding author(s): Shinichi Morishita

Last updated by author(s): Aug 4, 2023

# Reporting Summary

Nature Portfolio wishes to improve the reproducibility of the work that we publish. This form provides structure for consistency and transparency in reporting. For further information on Nature Portfolio policies, see our Editorial Policies and the Editorial Policy Checklist.

## Statistics

For all statistical analyses, confirm that the following items are present in the figure legend, table legend, main text, or Methods section.

| n/a | Confirmed | |
|---|---|---|
| ☐ | ☒ | The exact sample size (*n*) for each experimental group/condition, given as a discrete number and unit of measurement |
| ☐ | ☒ | A statement on whether measurements were taken from distinct samples or whether the same sample was measured repeatedly |
| ☐ | ☒ | The statistical test(s) used AND whether they are one- or two-sided<br>*Only common tests should be described solely by name; describe more complex techniques in the Methods section.* |
| ☐ | ☒ | A description of all covariates tested |
| ☐ | ☒ | A description of any assumptions or corrections, such as tests of normality and adjustment for multiple comparisons |
| ☐ | ☒ | A full description of the statistical parameters including central tendency (e.g. means) or other basic estimates (e.g. regression coefficient) AND variation (e.g. standard deviation) or associated estimates of uncertainty (e.g. confidence intervals) |
| ☐ | ☒ | For null hypothesis testing, the test statistic (e.g. *F*, *t*, *r*) with confidence intervals, effect sizes, degrees of freedom and *P* value noted<br>*Give P values as exact values whenever suitable.* |
| ☒ | ☐ | For Bayesian analysis, information on the choice of priors and Markov chain Monte Carlo settings |
| ☐ | ☒ | For hierarchical and complex designs, identification of the appropriate level for tests and full reporting of outcomes |
| ☐ | ☒ | Estimates of effect sizes (e.g. Cohen's *d*, Pearson's *r*), indicating how they were calculated |

*Our web collection on statistics for biologists contains articles on many of the points above.*

## Software and code

Policy information about availability of computer code

| | |
|---|---|
| Data collection | *Provide a description of all commercial, open source and custom code used to collect the data in this study, specifying the version used OR state that no software was used.* |
| Data analysis | Codes are available at https://github.com/morisUtokyo/cTR (DOI: 10.5281/zenodo.8207183), https://github.com/morisUtokyo/uTR (DOI: 10.5281/zenodo.8207190), and https://github.com/morisUtokyo/hTR (DOI: 10.5281/zenodo.8207188). Supplementary Figure 8 is a flowchart outlining how these programs are used in the various analysis stages. To analyze the data, we used R version 4.0.3, Tandem Repeats Finder (TRF), version 4.09, mTR (https://github.com/morisUtokyo/mTR), ExpansionHunter version 4.0.1, and minimap2 version 2.13. |

For manuscripts utilizing custom algorithms or software that are central to the research but not yet described in published literature, software must be made available to editors and reviewers. We strongly encourage code deposition in a community repository (e.g. GitHub). See the Nature Portfolio guidelines for submitting code & software for further information.

## Data

Policy information about availability of data

All manuscripts must include a data availability statement. This statement should provide the following information, where applicable:

- Accession codes, unique identifiers, or web links for publicly available datasets
- A description of any restrictions on data availability
- For clinical datasets or third party data, please ensure that the statement adheres to our policy

All sequencing data and TR loci for 270 Japanese samples are deposited in the NBDC Human Database under Data Set ID JGAS000286 [https://humandbs.biosciencedbc.jp/en/hum0174-v4#JGAS000286] and JGAS000505 [https://humandbs.biosciencedbc.jp/en/hum0174-v5#JGAS000505], and are available under restricted access for preservation of confidentiality of personal data. Access can be obtained by a direct application for using NBDC Human Data (see the details in https://humandbs.biosciencedbc.jp/en/data-use).

As the reference human genome, hg38 was used.

Extended Data Table 1 shows TR loci such that the longest TRs were >100 b longer than the median. If a TR locus in a gene coding region, we annotated it with the gene name and its location within the gene (exon, intro, UTR, etc) using the UCSC hg38 tables:
https://hgdownload.soe.ucsc.edu/goldenPath/hg38/database/kgXref.txt.gz
https://hgdownload.soe.ucsc.edu/goldenPath/hg38/bigZips/genes/hg38.knownGene.gtf.gz
We used geneSymbol in the first table above if it was present and spID in the second table otherwise. When a TR overlaps with untranslated regions (UTR) and exons, it is treated as being associated with UTR. When TR overlaps with an exon but does not any UTR, it is associated with the exon. When a TR is properly included in an intron, it is labelled with the intron.

We downloaded the following well-annotated GnomAD table of 60 disease-associated TR regions that were generated from short read sequencing data by ExpansionHunter v4.0.1:
https://gnomad.broadinstitute.org/short-tandem-repeats?dataset=gnomad_r3

## Research involving human participants, their data, or biological material

Policy information about studies with human participants or human data. See also policy information about sex, gender (identity/presentation), and sexual orientation and race, ethnicity and racism.

| | |
|---|---|
| Reporting on sex and gender | Because of the need to profile the distribution of tandem repeats of the Y human chromosome, 258 of the 270 analyzed samples were male and 10 were female. |
| Reporting on race, ethnicity, or other socially relevant groupings | No grouping is done for this item. |
| Population characteristics | We used immortalized B cells derived from Japanese subjects that were distributed by Japanese Collection of Research Bioresources (Japanese B cell DNA bank), the National Institute of Biomedical Innovation, Health and Nutrition. We selected 270 samples at random to characterize the distribution of structural variants (mosaic tandem repeats). |
| Recruitment | Participants are recruited by Japanese Collection of Research Bioresources (Japanese B cell DNA bank), the National Institute of Biomedical Innovation. They have been distributing cell resources to researchers around the world since 1985. |
| Ethics oversight | This study was approved by the Research Ethics Committee of the Faculty of Medicine of the University of Tokyo (Human Genome/Gene Analysis Research Ethics Review; review no. 19-323). |

Note that full information on the approval of the study protocol must also be provided in the manuscript.

# Field-specific reporting

Please select the one below that is the best fit for your research. If you are not sure, read the appropriate sections before making your selection.

☒ Life sciences ☐ Behavioural & social sciences ☐ Ecological, evolutionary & environmental sciences

For a reference copy of the document with all sections, see nature.com/documents/nr-reporting-summary-flat.pdf

# Life sciences study design

All studies must disclose on these points even when the disclosure is negative.

| | |
|---|---|
| Sample size | Over 7.5 times the coverage of the PacBio HiFi reads was collected from each of the 270 samples, making this the largest collection of high precision reads to our knowledge. In the manuscript, we show that the dataset theoretically allows the observation of mosaic TRs with lengths of 5, 2, and 1 kb within one of two haplotypes with ≥ 1 read of 14 kb read at probability levels of 91%, 96%, and 97%, respectively, according to the Lander-Waterman statistics (Methods, Extended Data Fig. 2). We understand that the probability can be improved by collecting more data, but this will be an issue for the future. |

| | |
|---|---|
| Data exclusions | No data are excluded. |
| Replication | The validity of the computational, experimental results is confirmed by statistical tests (not by replication tests). |
| Randomization | 270 samples were randomly selected from B cells derived from Japanese subjects that were distributed by Japanese Collection of Research Bioresources (Japanese B cell DNA bank), the National Institute of Biomedical Innovation, Health and Nutrition. Sample allocation was random. |
| Blinding | The investigators were blinded to group allocation during data collection and/or analysis. Blinding has been already conducted at Japanese Collection of Research Bioresources (Japanese B cell DNA bank), the National Institute of Biomedical Innovation, Health and Nutrition. |

# Reporting for specific materials, systems and methods

We require information from authors about some types of materials, experimental systems and methods used in many studies. Here, indicate whether each material, system or method listed is relevant to your study. If you are not sure if a list item applies to your research, read the appropriate section before selecting a response.

## Materials & experimental systems

| n/a | Involved in the study |
|---|---|
| ☒ | ☐ Antibodies |
| ☒ | ☐ Eukaryotic cell lines |
| ☒ | ☐ Palaeontology and archaeology |
| ☒ | ☐ Animals and other organisms |
| ☒ | ☐ Clinical data |
| ☒ | ☐ Dual use research of concern |
| ☒ | ☐ Plants |

## Methods

| n/a | Involved in the study |
|---|---|
| ☒ | ☐ ChIP-seq |
| ☒ | ☐ Flow cytometry |
| ☒ | ☐ MRI-based neuroimaging |
